# Tropomyosin: An Excretory/Secretory Protein from *Haemonchus contortus* Mediates the Immuno-Suppressive Potential of Goat Peripheral Blood Mononuclear Cells In Vitro

**DOI:** 10.3390/vaccines8010109

**Published:** 2020-03-01

**Authors:** Muhammad Ehsan, Muhammad Haseeb, Ruisi Hu, Haider Ali, Muhammad Ali Memon, Ruofeng Yan, Lixin Xu, Xiaokai Song, Xingquan Zhu, Xiangrui Li

**Affiliations:** 1 MOE Joint International Research Laboratory of Animal Health and Food Safety, College of Veterinary Medicine, Nanjing Agricultural University, Nanjing 210095, Jiangsu, China; mehsan124@gmail.com (M.E.); muhammadhaseeb73@gmail.com (M.H.); 2018207074@njau.edu.cn (H.A.); 2016207040@njau.edu.cn (M.A.M.); yanruofeng@njau.edu.cn (R.Y.); xulixin@njau.edu.cn (L.X.); songxiaokai@njau.edu.cn (X.S.); 2 State Key Laboratory of Veterinary Etiological Biology, Key Laboratory of Veterinary Parasitology of Gansu Province, Lanzhou Veterinary Research Institute, Chinese Academy of Agricultural Sciences, Lanzhou 730046, Gansu, China; grishu0707@gmail.com (R.H.); zhuxingquan@caas.cn (X.Z.)

**Keywords:** *Haemonchus contortus*, tropomyosin, goat, PBMCs, host-parasite interactions, immune responses

## Abstract

During host-parasite interactions, binding of excretory/secretory proteins (ESPs) on the host immune cells is considered the fundamental phase for regulation of immune responses. In this study, gene encoding *Haemonchus contortus* tropomyosin (Hc-TpMy), was successfully cloned and expressed, and the recombinant protein after host cell surface attachment was evaluated for immune functional analysis with goat peripheral blood mononuclear cells (PBMCs) in vitro. The isopropyl-β-D-thiogalactopyranoside (IPTG)-induced recombinant protein was successfully recognized by the sera of rat experimentally infected with rHc-TpMy. The immunofluorescence assay detected attachment of rHc-TpMy on the surface of host PBMCs. Furthermore, immunoregulatory roles of rHc-TpMy on cytokines expression, PBMC proliferation, migration, nitric oxide (NO) production, apoptosis and monocytes phagocytosis were observed. The results showed that expression of IL-4 and IFN-γ cytokines, cell proliferation, NO production and PBMC migration were significantly suppressed by goat PBMCs after co-incubation with rHc-TpMy protein. However, the productions of IL-10, IL-17 and TGF-β1 cytokines, PBMCs apoptosis and monocytes phagocytosis were elevated at dose dependent manner. Our findings indicated that rHc-TpMy is an important ES binding protein exhibit distinct immuno-suppressive roles on goat PBMCs which might be a potential molecular target to control haemonchosis in future.

## 1. Introduction

The gastrointestinal nematode parasite *Haemonchus contortus* is a serious worldwide problem, particularly in the goat and sheep industry, where it causes mortality in young animals due to its blood feeding behavior in abomasal mucosa [1]. The existing control strategies for nematode infection through widespread use of antihelmintics have resulted in serious drug resistance in domestic livestock [2,3]. The emergence of drug resistance demands for novel anti-parasitic drugs and vaccines, and development of powerful immunological approaches to control nematode infections [4]. Therefore, a deep insight into developmental biology of *H. contortus* at a molecular level might determine some key antigens as new drug targets, which could provide promising vaccine candidates against haemonchosis.

The use of *H. contortus* excretory and secretory proteins (HcESPs)-induced protective immunity against parasite and recombinant antigens have been under extensive investigation as a control strategy against haemonchosis [5,6]. Helminth infections, including nematodes, are habitually associated with increased IgE levels and type I hypersensitivity reactions, which in turn are responsible for the expression of interleukins and T helper cell type 2 (Th2) immune responses induced by ES antigens [7]. During nematode infections, a battle for survival and existence occurs, where hosts mainly rely on Th2 cells associated with type 2 immune responses [8,9], responsible for the secretion of various cytokines [10] and B cell differentiation, and expression of antibodies and migration of eosinophils at the target site to remove parasites [11], while, on the other hand, Th2 immune responses are also a big challenge for worm survival by minimizing Th1-based inflammatory responses/pathology and maintain the stability of the immune system by providing a better optimized homeostatic environment [12]. 

Tropomyosin belongs to a family of highly conserved proteins, which maintain rigidity and stability in both muscle and non-muscle cells by regulating actin filaments’ function by myosin and troponin [13]. Structurally and functionally, tropomyosin exhibits highly conserved sequences among nematodes, and because of its antigenic nature it is expressed by every cell during infection. Tropomyosin is distributed almost in all eukaryotic organisms and also found in multiple isoforms depending on the type of tissue [14]. Moreover, several T cell and B cell epitopes, distributed at various protein binding regions, induce strong T and B cell responses [15,16]. Previously, it was suggested that tropomyosin, with twenty different isoforms, has been involved in distinct cellular functions such as cytokine secretion, intracellular transport, cell transformation, cell proliferation and movement [17,18,19]. 

In our previous research, tropomyosin was identified as an excretory and secretory protein (ESP) that binds to goat PBMCs at the L4 and L5 developmental stages of *H. contortus* [20], and we also demonstrated that HcESPs exhibit suppressive regulatory roles in their interaction with PBMCs [21]. In our recent study, recombinant tropomyosin was found to have high immunodiagnostic potential for early stage diagnosis of *H. contortus* infection in goats [22]. However, the immunological functional impacts of Hc-TpMy on goat PBMCs remained unknown. In this study, we cloned a *H. contortus* tropomyosin (Hc-TpMy) gene, and the recombinant protein was expressed and purified to evaluate its immunological impact on goat PBMCs in vitro. This might be helpful to understand the development biology and effective immune invasion mechanism of *H. contortus* during host-parasite interactions. 

## 2. Material and Methods

### 2.1. Animals and Parasites

Helminth-free local crossbred goats (6 month to 1 year old) were kept indoors under controlled conditions at the Nanjing Agricultural University, and provided with hay, whole shelled corn and water *ad libitum*. The goats were maintained in helminth-free conditions and dewormed at two weeks intervals with the anthelminthic drug levamisole (8 mg/kg body weight) administered orally [23]. Fecal samples were subjected to regular microscopic examination for helminth eggs and the health of animals was monitored during the whole study period. 

Nanjing (2005) strain *H. contortus* nematodes were isolated as detailed in a previous study [24] and maintained in one year old helminth-free goats. From eggs collection and culture to L3 stage larvae, experiments were carried out as described previously [25,26]. A bolus containing ~8000 *H. contortus* L3 larvae were fed orally to the goats. Infection was checked on a weekly basis by microscopic examination and at 28 days post-infection, the donor goats were euthanized, slaughtered and adult worms were collected from the abomasum, and stored in liquid nitrogen for further study. Three biological replicates (three goats), each consisting of three technical replicates (three replicates for each goat), were run for immune and functional studies including immunofluorescence assays, cytokine expression analysis, cell proliferation, nitric oxide production, migration assay and apoptotic activity. Sprague Dawley (SD) rats about ~150g body weight, were purchased from the Experimental Animal Center of Jiangsu, PR China (Qualification Certificate: SCXK 2008-0004) and were reared in a microbe-free environment provided with sterilized food and water. 

### 2.2. PBMCs Separation and Culture

Whole blood was taken by venipuncture of the jugular vein, drained into 10 mL vacutainers coated with ethylenediaminetetraacetic acid (EDTA) and brought to laboratory for PBMCs isolation and culture. Peripheral blood mononuclear cells (PBMCs) were separated by Ficoll-hypaque (GE Healthcare, Madison, WI, USA) density gradient centrifugation protocol [27]. Following a washing step (three times) with PBS (Ca^2+^/Mg^2+^-free pH 7.4) cell viability was assessed by a Trypan Blue exclusion test for consistency >95%. Cells with a final density at 1 × 10^6^ cells/mL were incubated with Roswell Park Memorial Institute (RPMI) 1640 culture media containing 10% heat-inactivated fetal bovine serum (FBS) (ThermoFisher, Waltham, MA, USA), 100 U/mL penicillin and 100 mg/mL streptomycin (GIBCO, Grand Island, New York, USA). For functional study, PBMCs were cultured in 24-well flat-bottomed culture plates (Costar, Cambridge, MA, USA) with varying concentrations of rHc-TpMy at 37 °C and 5% CO_2_ for different time periods according to the test required.

### 2.3. RNA Isolation and Construction of cDNA Encoding H. contortus TpMy 

*H. contortus* adult worms stored in liquid nitrogen were taken out and subjected to RNA extraction followed by cDNA synthesis. RNA was isolated under RNase free condition using Trizol (Invitrogen, Carlsbad, CA, USA) according to the manufacturer’s instructions. Briefly, the worms in pre-chilled pestle and mortar containing 1 ml Trizol were minced and homogenized for 30 minutes followed by addition of 200 µL of trichloromethane and centrifugation at 10, 000× *g* for 15 min at 4 °C. Supernatant was precipitated by addition of 0.25 volumes of isopropyl alcohol per 1 mL of Trizol and incubated at −20 °C for 30 min. RNA was pelleted at 10,000× *g* at 4 °C for 10 min. Pellet was dried and washed with 70 % ethanol and suspended in diethyl pyrocarbonate (DEPC) water. First strand cDNA was synthesized using reverse transcriptase cDNA Kit (TaKaRa Biotechnology, Dalian, China) according to the manufacturer’s instructions. The reaction was carried out with 3.5 µL of dNTP mixture (10 mM) and 1.5 µL oligo dT primers, and was run under two different temperatures at 70 °C for 10 min and 42 °C for 5 min and then cold on ice for 2 min. The final concentration was adjusted and stored at −20 °C until use.

### 2.4. Cloning and Expression of H. contortus TpMy Gene 

An open reading frame (ORF) of *H. contortus* tropomyosin (GenBank/Uniprot: HF965396/ U6PU35), was amplified from cDNA by reverse transcription-polymerase chain reaction (RT-PCR) using specific pair of primers (sense 5′- GC**GGATCC**ATGTCGAAAGTGAACAAA - 3′- antisense 5′- GCG**AAGCTT**TCAATA- GCCGGACAGTTC -3′). The PCR amplification reactions of 50 µL total volume, contained 2 µL cDNA, 1.0 U Taq DNA polymerase (TaKaRa Biotech), 3.0 mM MgCl_2_, 400 µM dNTP mixture, 50 µM 10 × PCR Buffer (Mg^2+^-free) and 400 nM of each primer. The cycling parameters were: 94 °C for 5 min followed by 40 cycles of 94 °C 45 s, 55 °C 45 s, 72 °C for 1 min and final extension at 72 °C for 8 min.

The amplified PCR product was cloned into pMD19T vector (Takara Biotechnology, Nanjing, China) followed by transformation into Trans5α competent cells (TransGen Biotechnology, Beijing, China). Recombinant pMD19T-Hc-TpMy plasmid was confirmed by endonuclease restriction enzymes digestion. The purified product containing gene of interest was sub-cloned into the prokaryotic expression vector pET-32a (+) (Novagen, Madison, WI, USA) and transformed into BL21 (DE3) *Escherichia coli* cells (Novagen). Multiple colonies were picked, grown in LB media and amplified by PCR followed by restriction enzyme digestion with *BamH* I and *Hind* III. The recombinant plasmid pET-32a-Hc-TpMy was confirmed by sequence analysis with available sequences in GenBank databases using blast system (http://www.ncbi.nlm.nih.gov/BLAST/) for its exact insertion in reading frame. 

### 2.5. Sequence Analysis of Hc-TpMy 

The sequence identity of Hc-TpMy with known tropomyosin sequences available from the National Center for Biotechnology Information (NCBI) was analyzed by BLASTx and BLASTp (http://www.blast.ncbi.nlm.nih.gov/blast.cgi). The amino acid sequences from different nematode species were aligned and compared using ClustalW v1.8 software (http://www.clustal.org/). The phylogenetic tree was constructed using the Neighbor-Joining method and visualized by Molecular Evolutionary Genetics Analysis (MEGA 6.0 program; http://www.megasoftware.net/). The protein sequence was used to predict N-terminal signal peptides (http://www.cbs.dtu.dk/services/SignalP/), GPI modification Site Prediction (http://mendel.imp.ac.at/sat/gpi/gpiserver.html), T cell motifs (DNAstar: (EditSeq, Protean), Madison, WI, USA), B cell epitopes (http://tools.immuneepitope.org/tools/bcell/iedb input) as well as membrane protein prediction (http://www.cbs.dtu.dk/services/TMHMM/) by using bioinformatics approaches.

### 2.6. Expression and Purification of H. contortus TpMy Protein 

The recombinant plasmid pET-32a-Hc-TpMy was cultured in freshly prepared Luria Bertani (LB) medium containing tryptone (10 g/L), Nacl (10 g/L), yeast extract (5 g/L) (Oxoid™ Thermo Fisher Scientific, city, UK) and ampicillin (100 µg/mL) (Gold Biotechnology, St. Louis, MO, USA) at 37 °C, until the OD_600_ of the culture reached 0.5-0.7 at 37 °C. The protein expression was induced by 1 mM isopropyl-β-D-thiogalactopyranoside (IPTG; Sigma-Aldrich, Shanghai, China) at 37 °C for 7 h, and then collected by centrifugation. The pellet was lysed using lysozyme (10 µg/mL) (Sigma-Aldrich) followed by sonication and analyzed on 12 % (w/v) sodium dodecyl sulfate polyacrylamide gel electrophoresis (SDS–PAGE). The recombinant protein were purified by Ni^2+^-nitrilotriacetic acid (Ni-NTA) column (GE Healthcare, Madison, WI, USA) according to manufacturer’s instructions. The purification steps were followed as described previously [28]. Purity of the rHc-TpMy protein was determined by 12 % SDS-PAGE followed by coomassie blue staining. The concentration of recombinant proteins was checked by Bradford method [29], and stored at −20 °C for up streaming study. The fusion protein pET-32a with the 109aa Trx•Tag^TM^ thioredoxin protein and six histidines was obtained in a same way as rHc-TpMy and was used in downstream immunological study as a positive control.

### 2.7. Antibodies Production and Immunobloting

About 0.3 mg of the purified rHc-TpMy protein was mixed with equal volume of Freund’s complete adjuvant and injected subcutaneously into SD rats at multiple sites [30]. After two weeks, a booster dose with same volume of Freund’s incomplete adjuvant and rHc-TpMy protein was given through same route. Then rats were re-boosted three times at 1 week interval. After 10 days of last dose, the sera from immunized rats and normal rats (control) were collected and stored until used.

The resolved gel at 12 % SDS-PAGE, was transferred to polyvinylidene difluoride membranes (ImmobilonP, Millipore, Billerica, MA, USA) for immuno-blot analysis [31] using semi-dry system (Novablot, Hoefer, LV, USA) in transfer buffer (39 mM glycine, 48 mM Tris, 0.0375 % SDS, 20 % methanol) at 1.1 mA/cm^2^ for 1 h. Non-specific binding were blocked by submerging the membranes in 5 % (w/v) skimmed milk dissolved in Tris-buffered saline (TBS). The membranes were washed with TBS containing 0.1 % Tween-20 (TBST) and subsequently incubated with rat anti-rHc-TpMy as primary antibody (1:100 dilutions in TBST) at 4 °C overnight. After three washings with TBST, membranes were incubated with secondary antibody (1:3000 dilutions in TBST) horseradish peroxidase (HRP)-conjugated goat anti-rat IgG (Santa Cruz Biotechnology, Dallas, TX, USA) for 2 h at 37 °C. The membranes were washed and immunoreaction was visualized with diaminobenzidine (DAB, Sigma, St. Louis, MO, USA) within 5 min.

To detect native TpMy protein, adult *H. contortus* parasites were washed in pre-chilled phosphate buffered saline (PBS: Ca^2+^/Mg^2+^-free; pH 7.4) and disrupted on ice by adding radioimmunoprecipitation assay (RIPA) lysis buffer (Boster Biotechnology, Pleasanton, CA, USA). The supernatant was collected by centrifugation at ~10000 x g for 10 min and stored at −80 °C. Then lysates were electrophoresed on SDS-PAGE and transferred to the polyvinylidene difluoride (PVDF) membrane (Millipore). Hc-TpMy native protein was detected by westernblotting with rat anti-rHc-TpMy and goat anti-rat IgG (Southern Biotechnology, Birmingham, AL, USA) as first and second antibodies respectively, using the same procedure as above.

### 2.8. Binding of rHc-TpMy to Goat PBMCs

Detection of tropomyosin protein binding to PBMCs was performed according as described previously [32]. In detail, freshly 1×10^6^ cells were incubated with rHc-TpMy protein (treatment group) or pET32a empty protein (positive control group) and phosphate buffered saline (PBS) (negative control group) for 2 h at 37 °C with 5% CO_2_. After two successive washings, PBMCs were fixed on poly-L-lysine treated slides with 4 % paraformaldehyde in PBS for 30 min at room temperature. This was followed by blocking with 2 % bovine serum albumin (BSA) in PBS, cells were permeabilized with 1 % TritonX-100 and incubated with primary antibodies (1:100 dilution) rat anti-rHc-TpMy-O-IgG or normal rat sera (as control) for 4 h at 37 °C. Then cells were subjected to secondary antibody goat anti-rat IgG (1:1000 dilutions) coupled with Cy3 (Beyotime Biotechnology, Haimen, China) and maintained in dark with 2-(4-amidinophenyl)- 6-indolecarbamidine dihydrochloride (DAPI, 1.5 μM; Sigma). Slides were immersed in Anti-Fade Fluoromount solution (Beyotime Institute of Biotechnology, Nanjing, China) prior to visualization of protein binding under 100 × oil immersion on a laser scanning confocal microscope (LSM710, Zeiss, Jena, Germany). Digital images were captured using the Zeiss microscope software package ZEN 2012. 

### 2.9. ELISA Dependent Cytokines Secretion by PBMCs Treated With rHc-TpMy

The supernatants of cultured PBMCs, with 1 × 10^6^ cells density were used to determine cytokines level. Briefly, the cells were seeded in 24-well plates (1 ml/well) with serial concentrations of rHc-TpMy (10, 20, 40 and 80 µg/mL), pET32a protein (10 µg/mL) and equal volume of PBS (control). The mixtures were stimulated with 10 μg/mL of concanavalin A (ConA) (Sigma-Aldrich) for 72 h in RPMI 1640. Supernatants were collected by centrifugation at 200× *g* for 10 min and the concentration of interleukin-4 (IL-4), interleukin-10 (IL-10), interleukin-17 (IL-17), Interferon gamma (IFN-γ) and transformation growth factor-β1 (TGF-β1) were measured by commercially available goat ELISA kits (Jiancheng Biotechnology, Nanjing, China) [33] according to the manufacturer’s instructions. Each experiment was performed in triplicate.

### 2.10. Cell Proliferation Assay

Freshly isolated goat PBMCs (1 × 10^6^ cells/mL), with varying concentrations of rHc-TpMy (10, 20, 40 and 80 µg/mL), equal volume of control buffer (PBS) and pET32a protein were activated with ConA (10 μg/mL) for 72 h at 37 °C and 5% CO_2_. Cell proliferation tests were conducted as described by Gadahi et al. [20]. Cell counting kit-8 (CCK-8) reagent (10 µL/well) (Beyotime Biotechnology) was used 4 h prior to harvesting as per manufacturer’s instructions and absorbance was measured at 450 nm (OD_450_) using a microplate reader (BioRad Laboratories, Hercules, CA, USA). The OD_450_ value in control groups were set as 100 %. Cell proliferation index was calculated by the formula: OD_450_ of treatment/OD_450_ of control. Each experiment was performed in triplicate.

### 2.11. Cell Migration Assay

After incubation of PBMCs with varying concentrations of rHc-TpMy (10, 20, 40 and 80 µg/mL) along with recombinant protein of pET32a and equal volume of PBS (as control) for 24 h, the migration assay was performed as described earlier [34] using a Millicell^®^ insert with 8.0 μm pores (Merck Millipore, Darmstadt, Germany) according to the manufacturer’s instructions. Two hundred μL of cells (1× 10^6^ cells/mL) were seeded into the upper chamber and similarly the lower chamber was filled with 1300 μL RPMI 1640 medium and incubated for 2 h. The cells migrated through 8.0 µm pore size polycarbonate membrane into the lower chamber were determined by a Neubauer counting chamber. The results represents as mean percentage of seeded PBMCs. Each experiment was accomplished in triplicate.

### 2.12. Intracellular Nitric oxide Production 

The fresh separated PBMCs treated with or without rHc-TpMy as mentioned above were used to detect intracellular nitrite production by PBMCs using Griess assay [35] according to the instructions of Total Nitric Oxide Assay Kit (Beyotime Institute of Biotechnology) [36]. Absorbance values of the colored solution was measured using a plate reader (BioRad Laboratories) at 540 nm (OD_540_), and converted to micromoles per liter (μmol/L) using a standard curve that was generated by addition of 0 to 80 μmol/L sodium nitrite to fresh culture media. Individual experiments were performed in triplicate.

### 2.13. Cell Apoptosis Assay

The rHc-TpMy treated cells were subjected to apoptosis analysis as described by Li et al. [37]. According to manufacturer’s instructions of Annexin V-FITC kit (Miltenyi Biotechnology, Bergisch Gladbach, Germany), PBMCs (1.5 × 10^6^ cells/mL) were cultured with multiple concentrations of rHc-TpMy or pET32a protein and equal volume PBS for 24 h. The cells were washed twice with PBS (Ca^2+^/Mg^2+^-free, pH 7.4), re-suspended in binding buffer and apoptotic assay was performed as per kit instructions and checked on flow cytometry (BD Biosciences, San Jose, CA, USA). Results were analyzed at FlowJo 7.6 software (Tree Star, Ashland, OR, USA).

### 2.14. Statistical Analysis

The data were analyzed by using the GraphPad Prism v6.0 software package (Graphpad Inc., San Diego, CA, USA). The statistically significant differences among treatments (*p* < 0.05) were compared by one-way analysis of variance (ANOVA). Data were expressed as the mean ± the standard deviation (SD).

## 3. Results

### 3.1. Amplification, Cloning and Sequence Analysis of Hc-TpMy

The resultant fragment of 909 bp size was cloned into pMD19T vector and exact size was confirmed by sequencing. The cloned product inserted into prokaryotic expression vector pET32a-Hc-TpMy produced a fragment of about 909 bp, which was confirmed by restriction digestion with *BamH* I and *Hind* III. These results indicated that Hc-TpMy had been successfully inserted into frame of pET32a vector (Supplementary Materials Figure S1). 

Bioinformatics searching tools for similarity of Hc-TpMy to other known invertebrate tropomyosins revealed that Hc-TpMy sequence had 100% homology with *H. contortus* (CDJ92091), *Heligmosomoides polygyrus* (ABV44405) and *Teladorsagia circumcincta* (ADB27966), whereas, high similarity was found with *Trichostrongylus colubriformis* 99% (P15846), *Caenorhabditis elegans* 53% (NP_001300454), *Ascaris lumbricoides* 97% (ABS82498), *Anisakis simplex* 96% (Q9NAS5), *Heterodera glycines* 93% (AAQ12016) and *Dermatophagoides farinae* 85% (AIO08865). Contingency of the obtained sequence of Hc-TpMy was further analyzed by using multiple sequence alignment (ClustalW program) and phylogenetic relationship (MEGA 6.06 program). The phylogenetic analysis further confirmed that *H. contortus* tropomyosin protein was highly identical to the *H. contortus, Heligmosomoides polygyrus and Teladorsagia circumcincta* tropomyosin occupying same clusters as compared to other nematode parasites (Supplementary Materials Figure S2). However, no signal peptide was predicted in target sequence (Supplementary Materials Figure S3). 

### 3.2. Expression, Purification and Immunoblot of rHc-TpMy Protein

**Figure 1:**
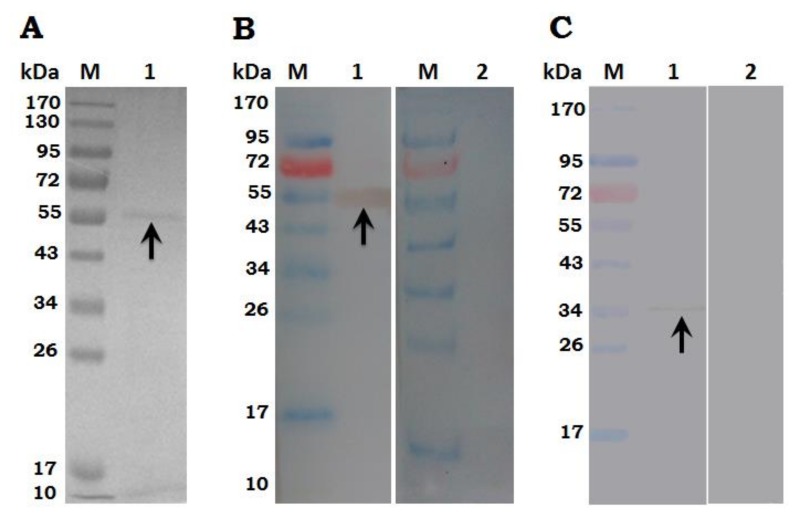
Purification and immuno blot analysis of rHc-TpMy protein. M: molecular weight standard protein Marker. **(A)** Lane 1: rHc-TpMy protein after isopropyl-ß-D-thiogalactopyranoside (IPTG) induction was purified by Ni-NTA column and resolved on SDS-PAGE. **(B)** M: molecular weight standard protein Marker; Lane 1: Purified rHc-TpMy was transferred to membrane and probed with serum from goat infected with *H. contortus*; Lane 2: membrane probed with normal goat serum as control. **(C)** M: molecular weight standard protein Marker; Lane 1: total ES proteins of *H. contortus* probed with antibodies from SD rats immunized with rHcMTF-12; Lane 2: Membrane probed with normal rat sera as control.

The rHc-TpMy expressed in *E. coli* BL21 strain, after induction by IPTG the protein expression was detected at different time interval and resolved at SDS-PAGE. The sonicated bacterial sediment purified by Ni-NTA super column indicated a single band of about 53 kDa on 12 % SDS-PAGE with fused pET32a protein of about 18 kDa (Figure 1A). The recombinant protein was consistent on the expected size of about 35 kDa after subtracting 18 kDa of fused vector protein. 

Immunoblot analysis showed that rHc-TpMy protein could be recognized by antibodies in sera from goats infected with *H. contortus* (Figure 1B). The native TpMy protein in whole soluble extracts of *H. contortus* was identified by sera from SD rats immunized with rHc-TpMy and a single band indicated that these antibodies had only specificity against Hc-TpMy protein of *H. contortus* (Figure 1C). However, no protein was detected with normal sera taken from un-immunized goats/rats.

### 3.3. Binding Confirmation of rHc-TpMy on Surface of Goat PBMCs 

**Figure 2:**
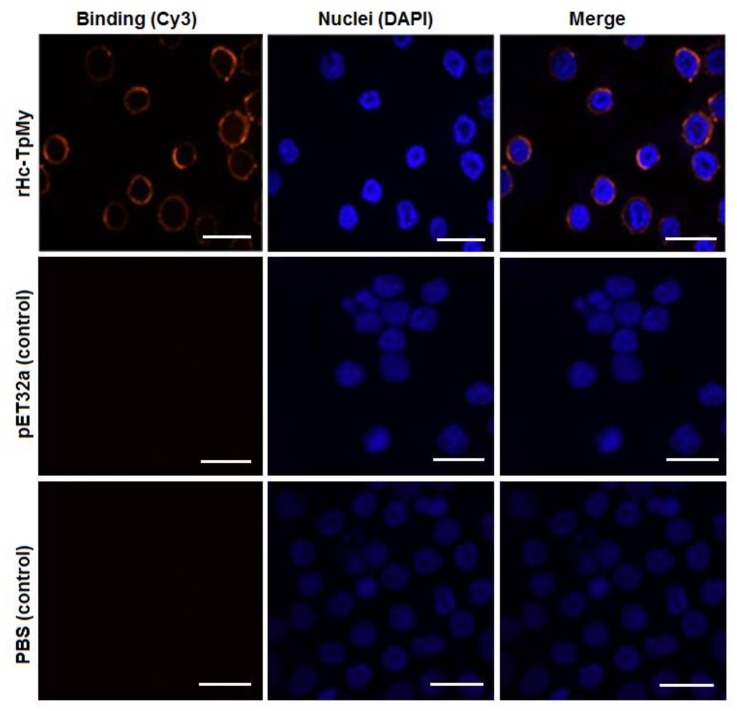
Identification of rHc-TpMy on surface of goat peripheral blood mononuclear cells PBMCs. PBMCs were cultured with rHc-TpMy (10 µg/mL), pET32a protein (10 µg/mL) or PBS as control at 37 °C for 2 h, and incubated with anti-rHc-TpMy, anti-pET32a protein or negative rat IgG (as first antibody), followed by staining with Cy3-conjugated secondary antibody (red). Nuclei were stained with DAPI (blue) and visualized at confocal laser scanning microscopy. Merge are the overlaps of red and blue channels. No red fluorescence was observed in control groups. Scale bar 10 µm.

The goat PBMCs cultured with rHc-TpMy and immunofluorescence assay using a confocal microscope imaging system revealed that rHc-TpMy protein could bind on surface of the goat PBMCs. As indicated in Figure 2, nuclei of the cells stained with blue florescence and target protein with red color. However, there was no florescence observed in control sections, either in PBS control or pET32a control group (Figure 2).

### 3.4. Cytokines Level in PBMCs Detected by ELISA 

**Figure 3:**
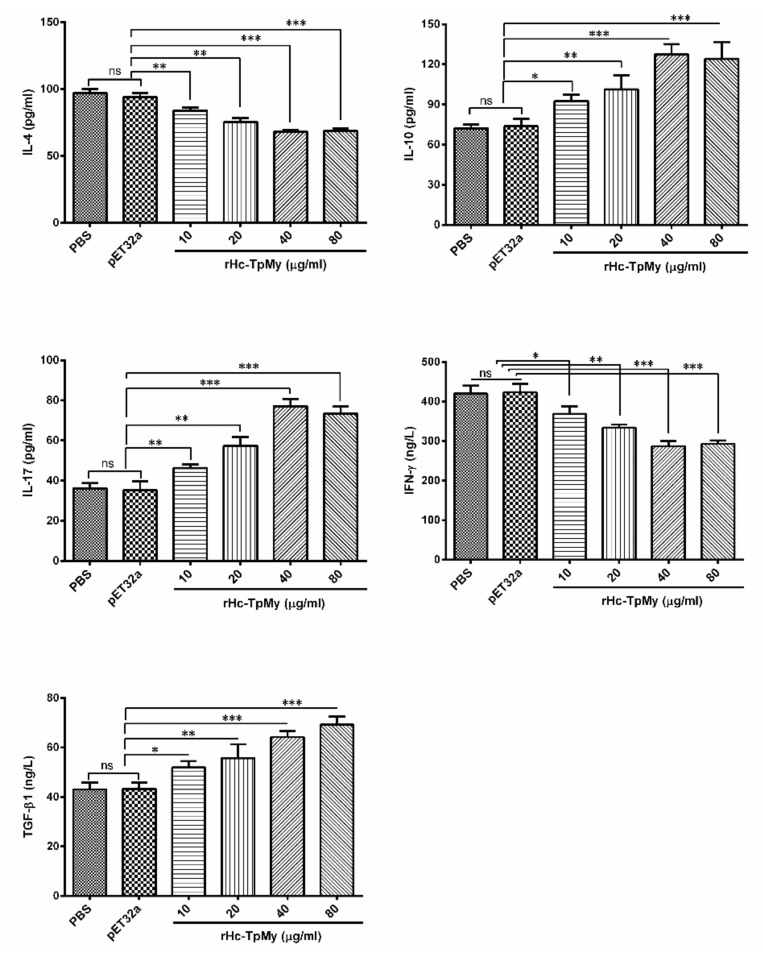
Patterns of cytokines expression in response to rHc-TpMy. PBMCs were stimulated with ConA (10 µg/mL) for 24 h in presence of rHc-TpMy or pET32a protein and PBS (control). Cytokines levels in supernatant of cell culture were quantified by ELISA kit. PBMCs used for all replicates of distinct treatments in each experimental repetition were derived from the same goat. The data are presented as the mean ± SD and are representative of three independent experiments (* *p* < 0.05, ** *p* < 0.01, ***p < 0.001, ns: non-significant).

The cytokines secretion level of goat PBMCs stimulated with ConA was measured by ELISA. The results of PBMCs treated with various concentrations of rHc-TpMy showed significantly decreased levels of IL-4 (ANOVA, *F*_(4, 10)_ = 30.81, *p* = 0.004) and IFN-γ (ANOVA, *F*_(4, 10)_ = 25.84, *p* = 0.001), whereas, IL-10 (ANOVA, *F*_(4, 10)_ = 17.54, *p* = 0.014), IL-17 (ANOVA, *F*_(4, 10)_ = 20.67, *p* = 0.001) and TGF-β1 (ANOVA, *F*_(4, 10)_ = 18.66, *p* = 0.001) expressions were increased at a dose dependent manner compared to that of control groups. No significant difference was observed in PBS control group and pET32a group (Figure 3).

### 3.5. PBMCs Proliferation Effected by rHc-TpMy

**Figure 4:**
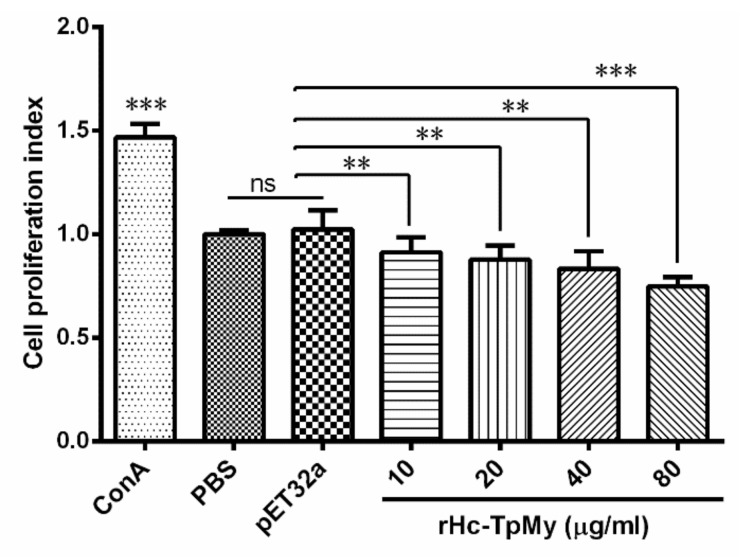
Influence of rHc-TpMy on PBMCs proliferation. Cells were stimulated with ConA and cultured with different concentrations of rHc-TpMy protein, pET32a protein or control buffer (PBS) at 37 °C and 5% CO_2_. The proliferation assay was determined by CCK-8 incorporation after 72 h. PBMCs used for all replicates of distinct treatments in each experimental repetition were derived from the same goat. The data are presented as the mean ± SD and representative of triplicate experiments (** *p* < 0.01 and *** *p* < 0.001, ns: non-significant).

The effect of rHc-TpMy on cell proliferation was measured by a cell counting kit (CCK8). Results highlighted that proliferation was significantly suppressed (ANOVA, *F*_(5, 12)_ = 13.43, *p* = 0.001) by the PBMCs incubated with 10 μg/mL (0.880 ± 0.072), 20 μg/mL (0.869 ± 0.046), 40 μg/mL (0.833 ± 0.049) and 80 μg/mL (0.748 ± 0.026) concentration of rHc-TpMy as compared to PBS control group (1.241 ± 0.061) and vector protein group (1.219 ± 0.076) (Figure 4). 

### 3.6. Cell Migration Assay

**Figure 5:**
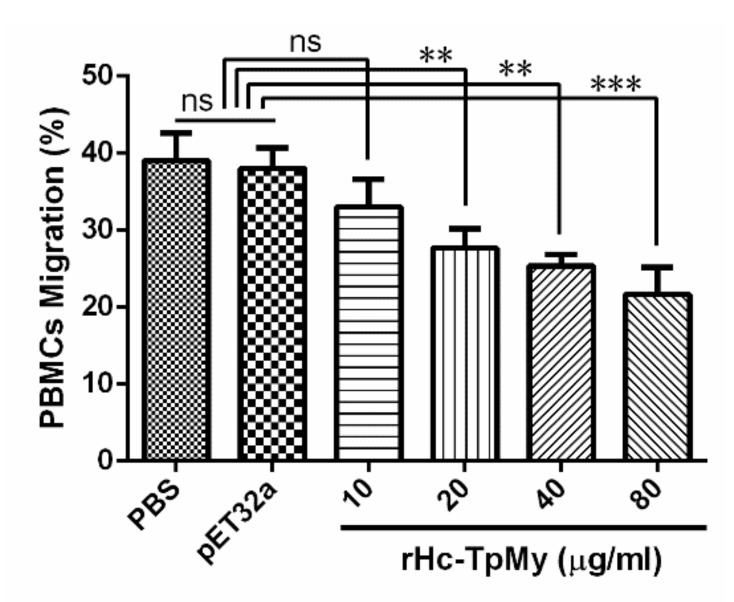
Inhibition effects of rHc-TpMy on cell migration. PBMCs with differential concentrations of rHc-TpMy protein, pET32a protein or control buffer (PBS) were stimulated and the random migration was determined. The difference between the mean values was calculated using ANOVA. PBMCs used for all replicates of distinct treatments in each experimental repetition were derived from the same goat. The data are presented as the mean ± SD and are representative of three independent experiments (** *p* < 0.01, *** *p* < 0.001 and ns: non-significant).

PBMCs migrated through the membrane in response to the rHc-TpMy was evaluated by using a Millicell^®^ insert (Merck Millipore). The percentage of migrated cells treated with rHc-TpMy at 20 µg/mL (27.67 ± 1.453 %), 40 µg/mL (25.33 ± 0.8819 %) and 80 µg/mL (21.67 ± 2.028 %) was gradually decreased (ANOVA, *F*_(5, 12)_ = 16.47, *p* = 0.004) in treated PBMCs (Figure 5), whereas, 10 µg/mL protein concentration showed no significant effect (35.00 ± 2.082 %) compared to that of PBS control (39.00 ± 2.082 %) and vector protein group (38.00 ± 1.528 %) group (ANOVA, *F*_(5, 12)_ = 16.47, *p* = 0.137) (Figure 5). 

### 3.7. Hc-TpMy Decreased NO Production in Goat PBMCs

**Figure 6:**
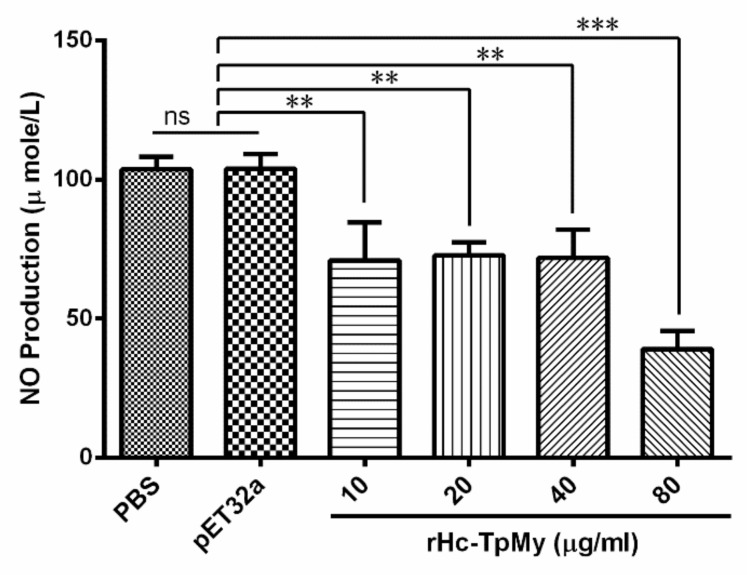
Impacts of rHc-TpMy on nitric oxide production. PBMCs were cultured with differential concentrations of rHc-TpMy protein, pET32a protein or control buffer (PBS), and intracellular nitrite concentration in the PBMCs was measured by using Griess assay. PBMCs used for all replicates of distinct treatments in each experimental repetition were derived from the same goat. The data are presented as the mean ± SD and representative of three independent experiments (** *p* < 0.01 and *** *p* < 0.001, and ns: non-significant).

Nitric oxide production by PBMCs treated with different concentrations of rHc-TpMy was measured by using the total nitric oxide assay kit. The results revealed that rHc-TpMy significantly suppressed the NO production (Figure 6). There was no differential regulation (ANOVA, *F*_(5, 12)_ = 26.25, *p* = 0.981) of NO between control sample (103.7 ± 2.653) and fused vector protein (103.8 ± 3.103) in response to Griess assay, whereas, NO production was significantly lowered (ANOVA, *F*_(5, 12)_ = 26.25, *p* = 0.001) with 10 µg/mL recombinant protein (70.91 ± 7.873), 20 µg/mL (72.66 ± 2.745), 40 µg/mL (71.84 ± 5.863) and 80 µg/mL (39.03 ± 3.805) as compared to control groups (Figure 6). 

### 3.8. Hc-TpMy Induced Apoptosis of PBMCs

**Figure 7:**
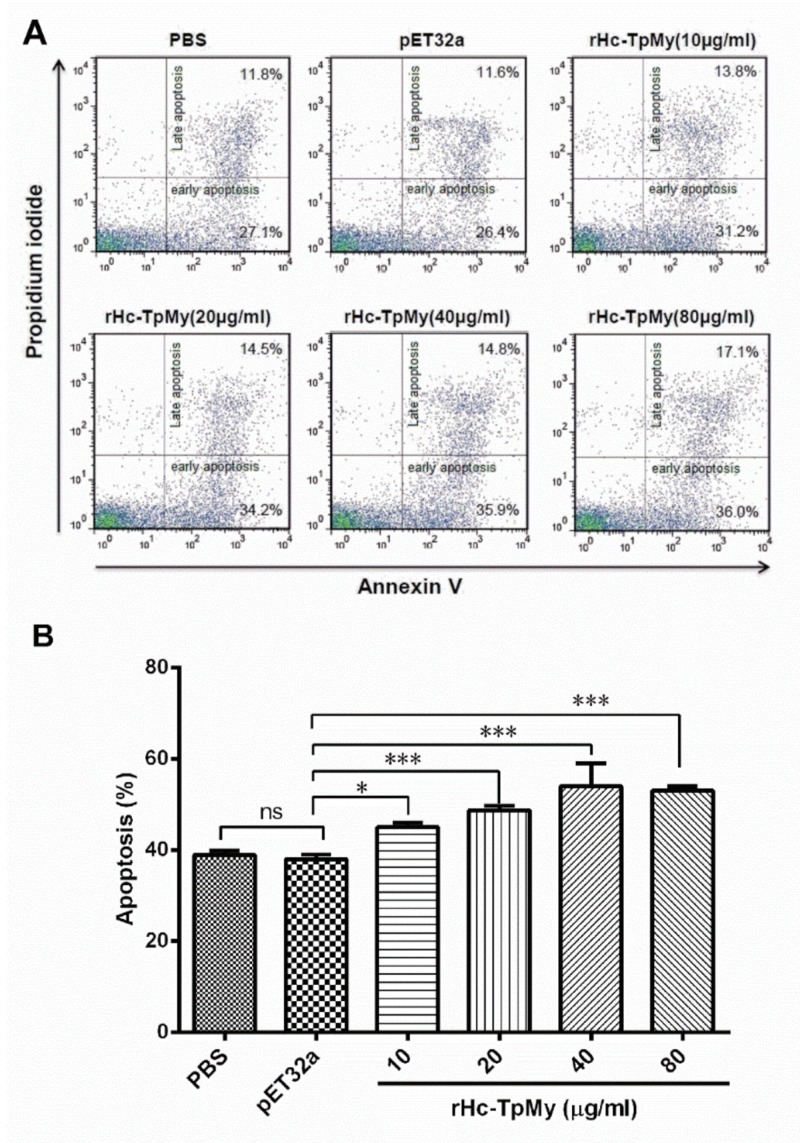
Apoptosis of goat PBMCs in respond to rHc-TpMy. **(A)** Apoptosis of PBMCs was determined by flow cytometry followed by staining with annexin V and PI. The percentages of cells with different staining patterns are shown. **(B)** rHc-TpMy increased apoptosis of goat PBMCs. The results are representative of three independent experiments. Data are presented as the mean ± SD (n = 3); an asterisk indicate treatment groups differ significantly (* *p* < 0.05) and highly significantly (*** *p* < 0.001) to that of control groups.

The discrete protein concentrations treated with goat PBMCs and their impacts on cell apoptosis were explored by apoptotic assay, using membrane phosphatidylserine (PS) as a marker of cell apoptosis and positive DNA staining as an indicator of membrane leakage. The results showed that increased protein concentration directed in upregulation of apoptosis percentage in goat PBMCs (ANOVA, *F*_(5, 12)_ = 28.94, *p* = 0.001; Figure 7). No significant difference in apoptosis rate was found between PBS (control) group (38.90 ± 0.577 %) and vector protein group (38.00 ± 0.577 %). However, rHc-TpMy-induced concentration-dependent apoptosis in PBMCs at 10 µg/mL (45.00 ± 0.577 %), 20 µg/mL (48.70 ± 0.5774 %), 40 µg/mL (54.03 ± 2.848) and 80 µg/mL (53.00 ± 0.577) as compared to control groups (Figure 7).

## 4. Discussion

Inside the host, *H. contortus* secrete and excrete various proteins that mediate modulatory or suppressive potential for immune responses during host parasite interactions. These responses are governed by the interaction of these proteins through receptor-ligand systems on the surface of host cells [30,38]. In our recent study, *H. contortus* tropomyosin was identified in excretory and secretory (ES) protein to bind to goat PBMCs *in vivo* at a specific developmental stage (L4 + L5) of the parasite [20], however, the host cells-related immunoregulatory functions affected by Hc-TpMy were not highlighted yet. In this study, we successfully cloned gene-containing *H. contortus* tropomyosin protein which elicited a suppressive regulatory role on goat PBMCs in vitro.

Tropomyosin is a well-known protein, that under non-reducing conditions shares different protein size patterns in filarial nematode codes, whereby a protein of 33 kDa shares 91 % similarity [39] whereas, *S. scabiei* encodes a band of 32.9 kDa with 98 % amino acid sequence homology with other vertebrates’ and invertebrates’ tropomyosin proteins [40]. In the case of *A. lumbricoides* and cockroach tropomyosin, the amino acid identity was more than 70 % to that of other invertebrates’ tropomyosins [41]. In the current study, we analyzed the protein sequence of Hc-TpMy and determined a 100 % resemblance with *H. contortus*, *T. circumcincta* and *H. polygyrus*. Tropomyosins of parasites have been considered as non-allergic, which was confirmed by detection of non-reactivity of sera from *Anisakis simplex*-affected individuals with native or recombinant tropomyosin of the same parasite [42]. Further studies supported this argument when a positive correlation was found between helminth infection and a low level of skin reactions against house-dust mite [43]. In this study specific antibodies against rHc-TpMy were detected in sera from experimental rats. To regulate immune functions effectively, (ESPs) requires receptor-ligand systems on the surface of the host cells [44]. Previously, we stated that a variety of interacting proteins termed as HcESPs are capable of binding with host immune cells and decreasing the immune functions significantly [21]. In the present study, we determined that a purified rHc-TpMy in interaction with goat PBMCs could attach on the surface of cells to regulate immune functions but the mechanism is governed by pathway(s) worthy of further investigation.

The immune (Th1 + Th2) and inflammatory responses against parasitic infection, especially by *H. contortus* are regulated by cells and cytokines produced by the host [8]. The resistance in sheep during parasitic infection is typically associated with expression of IL-4, which mediates a Th2 type immune response [45]. It was considered that IL-4 acts as a feedback for Th2 differentiation, regulated by IFN-γ [46]. It has been demonstrated that on stimulation of recombinant tropomyosin from *Acanthocheilonema vitae*, the IL-4 production by spleen cells was decreased simultaneously [47]. During helminth infection, IFN-γ produced by Th1 cells regulates cellular immunity, antigen presentation and protective immune responses [48]. In our previous study HcESPs and rHc-14-3-3 suppressed IL-4 and IFN-γ secretions by PBMCs in vitro [21,49]. Our data accord with earlier studies indicating that rHc-TpMy could inhibit the Th1- and Th2-based immune responses by decreasing IL-4 and IFN-γ production in PBMCs. IL-10 belongs to family of IL-10 cytokines initiated by T regulatory cells (T_reg_), which elicited a suppressive role on host immune responses and facilitate tissue repair processes during infection or inflammation, and are responsible for decreased expression of Th1 cytokines, MHC II antigens and co-stimulatory molecules of macrophages [50]. However, previously it was shown that production of IL-10 either by native or recombinant tropomyosin was associated with lower cell activity and downregulation of immune functions. Wilson and Maizels [51] reported that tropomyosin from helminths induced IL-10 expression that diminished the activation of anti-inflammatory responses and resulted in decreased immunological responses. In this study rHc-TpMy protein affected Th1 and Th2 immune responses by enhancing IL-10 production in goat PBMCs. Transforming growth factor-β (TGF-β1) is a multifunctional cytokine produced by T and B-cells, macrophages and variety of other cells, and involved in cell growth and differentiation [52]. This cytokine is potentially involved in different immunoregulatory activities, biological processes and immunosuppressive properties [53]. In the present study, the increased level of TGF-β1 in response to rHc-TpMy protein strongly supported the current findings and suggested that TpMy as an important constituent of HcESPs might contribute to immunosuppressive roles of HcESPs during host-parasite interactions. Interleukin-17 (IL-17) producing cells assumed to have distinct subset from Th1 and Th2 cells and functionally characterized as inflammatory modulators with a significant role in immune responses against various parasitic infections [54,55,56]. In our previous study, we found that *H. contortus* HcESPs collectively increased the IL-17 production [21], whereas, IL-17 levels were upregulated under the influence of rHcftt-2 on goat PBMCs [49]. In this research rHc-TpMy significantly increased the production of IL-17, which might create a favorable environment for parasite infection. The real effect of Th17 in the context of Th1 and Th2 immune responses needs to further elucidation.

In different species tropomyosins exhibit resemblances in functions and chemical properties, depending upon peptide sequences, antigenic structure and epitopes which varies from species to species [57]. Antigen-presenting cells (APCs) and T cells have an active role in modulating or decreasing immune cell proliferation, therefore they could alter immune responses [58]. In a shrimp model tropomyosin proteins and their derived peptides stimulated not only B cell responses, but also induced CD4^+^ T cell proliferation and responses, that lead to shift in cytokines secretion and Th2 responses [59]. We have reported that rHc-TpMy significantly suppressed the proliferation of goat PBMCs. These findings might be supportive for diminishing Th2 type cytokine secretions and immune responses by *H. contortus* tropomyosin. During helminth infections, parasites develop inflammatory responses, regulated primarily by chemokines or cytokines. The recruitment of effector cells (leukocytes, eosinophils and lymphocyte) to the site of infection and their functions serve for disease control [60]. Gastrointestinal nematodes could actively stimulate cell migration to the target area of infection resulting in inflammation, favorable for parasite existence [46]. In an earlier study, PBMCs treated with rHco-gal-m/f had a decreased trend of cell migration [30]. In our study rHc-TpMy actively downregulated the migration of goat PBMCs, which indicated that as an ES protein of *H. contortus* rHc-TpMy exhibited suppressive potential on PBMC migration. Nitric oxide produced by a number of immune cells, such as macrophages and non-immune-like hepatic and endothelial cells, in the context of regulation of some cytokines (IL4, 10, TGF-β1 and IFN-γ), is involved in non-specific protection against a variety of helminth parasites [61]. Our recent studies on regulation of NO in response to HcESPs and recombinant rHcftt-2 showed an inhibition effect on NO production by PBMCs [21,49]. In this study, we evaluated a negative potential of rHc-TpMy on NO production along with a decreased trend of IFN-γ, which might facilitate some suppressive roles of HcESPs on NO production. The molecular and cellular mechanisms of parasites are considered as an inducer of immune responses via an apoptosis mechanism/pathway [62]. It was reported that many proteins of the galectin family are involved in host cell apoptosis [63,64]. Recent studies have demonstrated that rHco-gal-m induced the apoptosis of goat PBMCs in vitro [30,65]. Our results indicated that an increased level of apoptosis of goat PBMCs incorporated with rHc-TpMy, is involved in regulation of apoptosis in a dose dependent manner. 

## 5. Conclusions

*H. contortus* TpMy gene was cloned and recombinant Hc-TpMy protein was expressed. Immunoblot analysis determined that rHc-TpMy protein is an active member of HcESPs which could be recognized by antibodies in sera from goats infected with *H. contortus* and native TpMy protein by sera from rats immunized with rHc-TpMy. These findings demonstrate that rHc-TpMy as an important constituent of HcESPs that exhibit a distinct immuno-suppressive potential on goat PBMCs in vitro. The interaction of rHc-TpMy with host PBMCs played crucial roles on the secretions of suppressive cytokine IL-10, inflammatory modulator IL-17 and anti-inflammatory cytokine TGF-β1. The suppressive activity was further noted with a decreased level of IL-4 and IFN-γ production in addition to downregulation of the chemical factor NO, cell proliferative efficiency and cell migratory activity, which ultimately increased apoptosis of goat PBMCs. In our recent study, TpMy was found to be an important indicator with high immunodiagnostic potential for early diagnosis of *H. contortus* infections [22]. However, further study is urgently needed to check the effectiveness of rHc-TpMy in the goat-infected model, which could help understand profoundly the molecular mechanism(s) of this protein during host-parasite interactions.

Our findings not only contribute to an in-depth understanding of the immune functionality of rHc-TpMy in host immune cells, but might also help to elucidate the immune evasion mechanisms during host–parasite interactions. These immuno-suppressive characteristics suggest that rHc-TpMy could be a potential vaccine candidate for therapeutic interventions against haemonchosis in the near future. 

## 6. Ethics Statement and Participation Approval 

All research experiments and animals used in this research were in accordance to the guidelines of Animal Ethics Committee, Nanjing Agricultural University, and by the recommendations of Animal Welfare Council of China. The experiment’s protocols used in this research were all approved by the Science and Technology Agency of Jiangsu Province, with approval ID YXK (SU) 2010–0005.

## Supplementary Materials

The following are available online at https://www.mdpi.com/2076-393X/8/1/109/s1, Figure S1: Cloning and expression of Hc-TpMy gene, Figure S2: Comparison of rHc-TpMy amino acid sequences to that of other nematode species available on GenBank database, Figure S3: N-terminal signal peptide prediction in rHc-TpMy protein.
