# Supplementary material for: Tropomyosin: An Excretory/Secretory Protein from Haemonchus contortus Mediates the Immuno-Suppressive Potential of Goat Peripheral Blood Mononuclear Cells In Vitro
Source: Vaccines (Basel). 2020 Mar 1;8(1):109. doi: 10.3390/vaccines8010109 (PMC7157511; doi:10.3390/vaccines8010109)
Supplement: Supplementary file 1 [file vaccines-08-00109-s001.pdf]

vaccines

Article

Tropomyosin: An Excretory/Secretory Protein from *Haemonchus contortus* Mediates the Immuno-Suppressive Potential of Goat Peripheral Blood Mononuclear Cells In Vitro

Muhammad Ehsan [1,2], Muhammad Haseeb [1], Ruisi Hu [2], Haider Ali [1], Muhammad Ali Memon [1], Ruofeng Yan [1], Lixin Xu [1], Xiaokai Song [1], Xingquan Zhu [2] and Xiangrui Li [1,*]

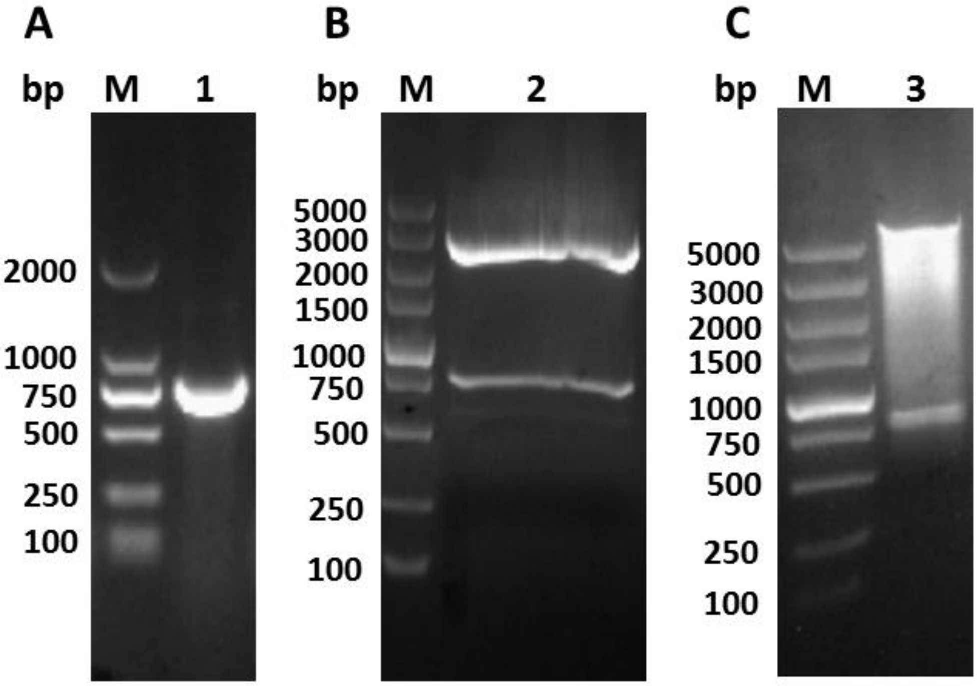

Figure S1. Cloning and expression of Hc-TpMy gene. (**A**) Gel electrophoresis of Hc-TpMy indicating 909 bp PCR product. (**B**) Amplified product cloned into pMD-19T vector. (**C**) Confirmation of recombinant expression plasmid pET32a verified by restriction digestion with *BamH* I and *Hind* III.

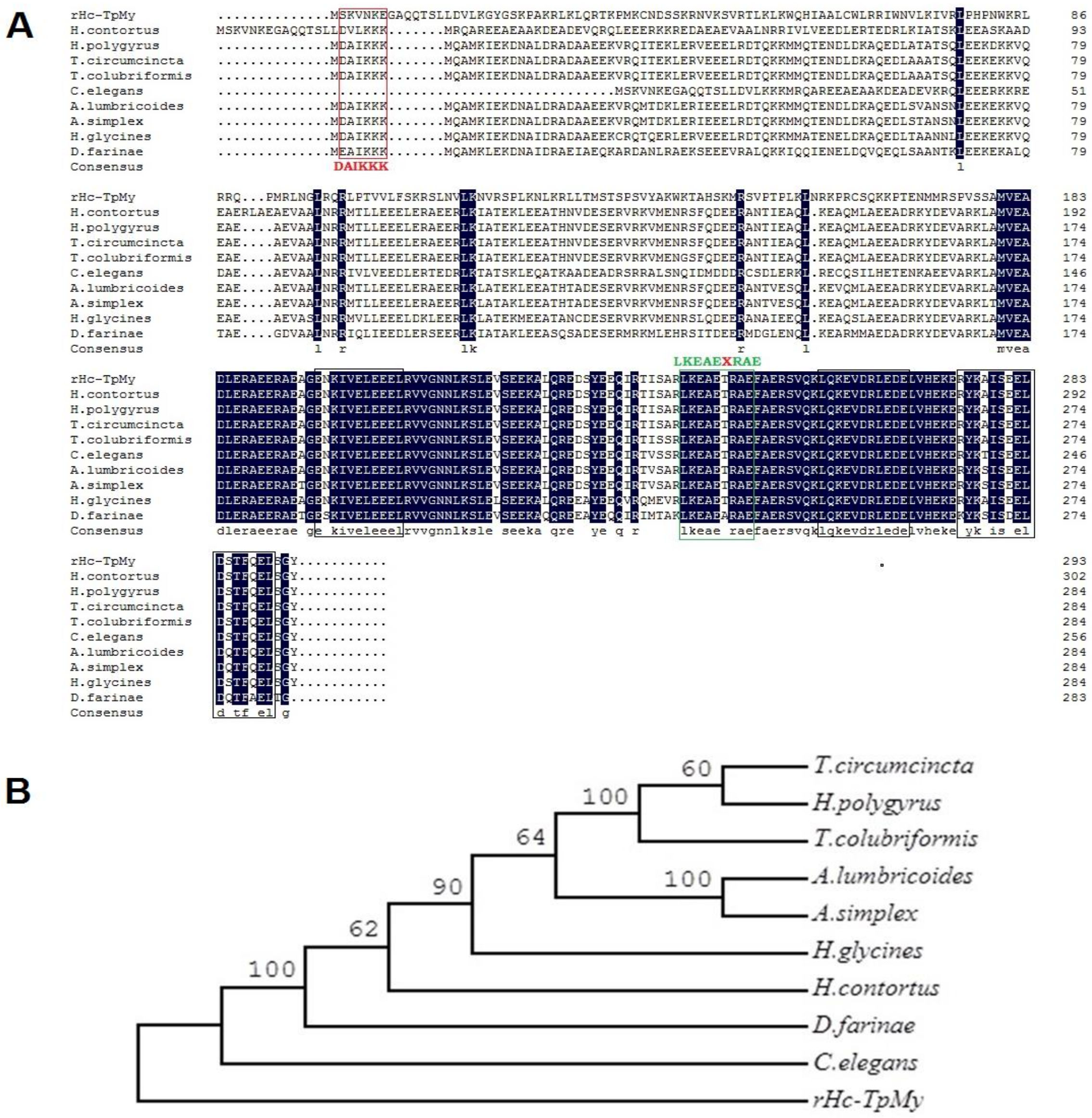

Figure S2. Comparison of rHc-TpMy amino acid sequences to that of other nematode species available on GenBank database. **(A)** *Haemonchus contortus* (CDJ92091), *Heligmosomoides polygyrus* (ABV44405), *Teladorsagia circumcincta* (ADB27966), *Trichostrongylus colubriformis* (P15846), *Caenorhabditis elegans* (NP_001300454), *Ascaris lumbricoides* (ABS82498), *Anisakis simplex* (Q9NAS5), *Heterodera glycines* (AAQ12016), *Dermatophagoides farinae* (AIO08865). The B cell epitopes of *H. contortus* tropomyosin to that of other sequences are shown in black boxes. Points indicate the identical residues with respect to the upper sequence (bold). **(B)** The kinship of Hc-TpMy to that of known nematodes tropomyosins available at GenBank database was evaluated by neighbour-joining method (1000 bootstrap) using MEGA ver. 6.0.

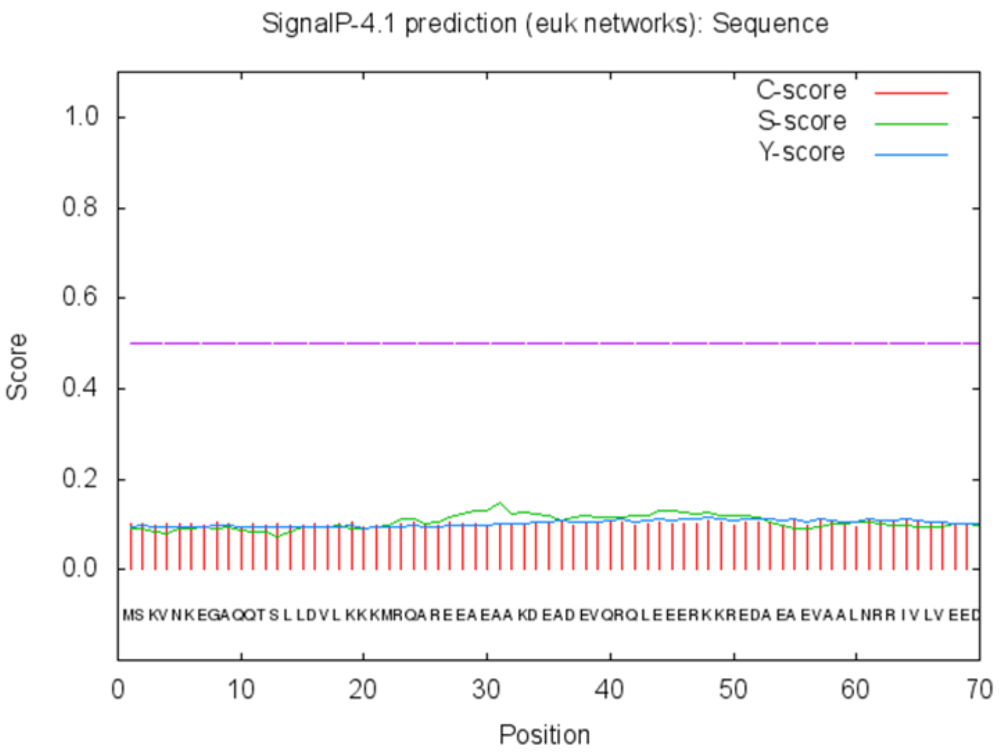

Figure S3. N-terminal signal peptide prediction in rHc-TpMy protein. The amino acid sequences of rHc-TpMy (NCBI accession numbers: HF965396) was used to predict N-terminal signal peptides by SignalP 4.1 Server (http://www.cbs.dtu.dk/services/SignalP/)**.**

© 2020 by the authors. Submitted for possible open access publication under the terms and conditions of the Creative Commons Attribution (CC BY) license (http://creativecommons.org/licenses/by/4.0/).
